# Cryopreservation of Canine Primary Dorsal Root Ganglion Neurons and Its Impact upon Susceptibility to Paramyxovirus Infection

**DOI:** 10.3390/ijms20051058

**Published:** 2019-02-28

**Authors:** Sarah Schwarz, Ingo Spitzbarth, Wolfgang Baumgärtner, Annika Lehmbecker

**Affiliations:** 1 Department of Pathology, University of Veterinary Medicine, 30559 Hannover, Germany; sarah.schwarz@tiho-hannover.de (S.S.); ingo.spitzbarth@tiho-hannover.de (I.S.); annika.lehmbecker@tiho-hannover.de (A.L.); 2 Center for Systems Neuroscience, 30559 Hannover, Germany

**Keywords:** canine distemper virus (CDV), cryopreservation, dorsal root ganglion (DRG) neurons, neuroinfection, paramyxovirus, canine, dog

## Abstract

Canine dorsal root ganglion (DRG) neurons, isolated post mortem from adult dogs, could provide a promising tool to study neuropathogenesis of neurotropic virus infections with a non-rodent host spectrum. However, access to canine DRG is limited due to lack of donor tissue and the cryopreservation of DRG neurons would greatly facilitate experiments. The present study aimed (i) to establish canine DRG neurons as an in vitro model for canine distemper virus (CDV) infection; and (ii) to determine whether DRG neurons are cryopreservable and remain infectable with CDV. Neurons were characterized morphologically and phenotypically by light microscopy, immunofluorescence, and functionally, by studying their neurite outgrowth and infectability with CDV. Cryopreserved canine DRG neurons remained in culture for at least 12 days. Furthermore, both non-cryopreserved and cryopreserved DRG neurons were susceptible to infection with two different strains of CDV, albeit only one of the two strains (CDV R252) provided sufficient absolute numbers of infected neurons. However, cryopreserved DRG neurons showed reduced cell yield, neurite outgrowth, neurite branching, and soma size and reduced susceptibility to CDV infection. In conclusion, canine primary DRG neurons represent a suitable tool for investigations upon the pathogenesis of neuronal CDV infection. Moreover, despite certain limitations, cryopreserved canine DRG neurons generally provide a useful and practicable alternative to address questions regarding virus tropism and neuropathogenesis.

## 1. Introduction

Primary neuronal cultures from rodents are an important tool for the investigation of cellular and molecular mechanisms of neurotropic virus infections [1,2]. Nevertheless, rodent cultures show limitations with respect to viruses that have a specific non-rodent host tropism. Moreover, rodent cultures do not always fully mirror the molecular characteristics of their human cellular counterparts. For instance, several investigations revealed that, regarding certain properties, canine glial cell preparations were more closely related to the human system than rodent ones [3,4]. Therefore, the dog represents a species with increasing importance as a translational animal model [5,6,7]. However, so far, few studies have investigated potential in vitro neuroinfectiology models in canines.

Canine distemper virus (CDV) is a paramyxovirus closely related to human measles virus and infects predominantly members of the order *carnivora* [8,9]. It can lead to persistent infection of the central nervous system (CNS) with a restrictive neuronal infection [10,11] that bears similarities with subacute sclerosing panencephalitis (SSPE), a devastating, late-onset neurological disease associated with human measles virus infection [8,12,13]. To enter epithelial cells, CDV employs the surface molecule nectin-4 [14], while signaling lymphocytic activation molecule (SLAM, CD150) represents its receptor on lymphocytes [15]. The receptor through which CDV enters neurons is so far unknown. Therefore a thorough investigation of CDV neuronal infection in vitro is of particular interest, especially considering that obtained results might potentially also be extrapolated to the infection of neurons by human measles virus. Two CDV strains were selected for the infection of canine DRG neurons in this study, which have previously been shown to infect central and peripheral canine primary glial cells in vitro to a different extent [16,17]. CDV R252 is a strain originally isolated from the spleen homogenate of an infected dog [18] and has been shown, next to its main manifestation of demyelinating leukoencephalomyelitis, to lead to gray matter disease in the CNS of dogs in vivo [19]. CDV-5804 PeGFP (eGFP = enhanced green fluorescent protein) has been produced out of a virus strain originally isolated from ferrets by the insertion of eGFP [20]. Due to its eGFP-expression it provides the advantage of direct observation of infected neurons in living cultures. Moreover, whether CDV-5804 PeGFP can infect canine neurons, in vivo or in vitro, is so far unclear, which makes it an interesting target for investigation.

The establishment and detailed morphological characterization of neuronal cultures of dogs represents a prerequisite for future research into viral neuropathogenesis. However, the cultivation of canine primary neurons from the brain has so far only been successful when using neonatal dogs, which have to be killed explicitly for this purpose. In addition, neuronal cells undergo rapid changes regarding the expression of neurotransmitter receptors and ion channels as well as the development of specific neuronal subpopulations during early neuronal development [21,22,23,24], indicating that fully differentiated neurons might be a more suitable tool to study diseases affecting mature animals. In contrast to neurons from the brain, dorsal root ganglion (DRG) neurons can be obtained from adult dogs, thus being fully differentiated. In addition, fresh post mortem tissue from adult dogs is sporadically available during routine necropsy or from control animals of unrelated studies. Therefore, cultivation of adult canine DRG neurons serves the principle of reducing, replacing and refining animal experiments (3R principle [25]).

Although rodent DRG neurons are already used on a regular basis in manifold studies including investigations upon toxicity, neurophysiology, neuropathic pain, and nociception [26,27,28,29,30], cultivation of canine DRG neurons has only relatively recently been established [31,32]. Although it is easier to obtain fresh post-mortem tissues from adult dogs compared to neonatal ones, their limited and sometimes unpredictable availability and the time-consuming isolation and culturing procedures of canine DRG neurons still present a challenge for their large-scale routine use in cell culture. Therefore, the establishment of a long-term storage protocol would be highly advantageous for future experiments. Cryopreservation of canine DRG-neurons would moreover allow repeated experiments with cells from the same source and establish a regularly available cell pool, consequently improving the planning and management of experiments including virus infection. Studies investigating the possibility of cryopreservation of primary canine DRG neurons are currently lacking. So far, most studies on the cryopreservation of primary nerve cells or tissue fragments have focused on fetal and neonatal rats or mice [33,34], cell-lines [35,36], or stem cells [37,38,39,40]. There are only very few studies on postnatal rats or mice [41,42,43,44]. Besides rodent neurons, cryopreservation has also been successfully applied to neurons isolated from fetal cynomolgus monkeys [45], sheep [46], bovines [47], and humans [48,49,50]. Although cryopreservation of cells of any kind naturally results in a loss of cell-yield and viability [51], the majority of these studies did not report a significant effect of cryopreservation upon neuronal morphology [38,41,45,48,52,53], electrophysiological properties [34,45], and neurite outgrowth behavior [34,41,44], thus indicating that cultivation of cryopreserved neuronal cells represents a promising approach.

Therefore, in order to set up a basis for future neuroinfectiology experiments using canine DRG neurons as a potentially promising in vitro model system, the present study aimed (i) to establish canine DRG neurons as an in vitro model for infection with CDV; and (ii) to determine whether DRG neurons are cryopreservable and remain infectable with CDV. 

## 2. Results

### 2.1. Non-Infected Cultures, Non-Cryopreserved and Cryopreserved: Neuronal Morphology, Cell Yield, Viability, and Cleaved Caspase 3-Expression

**Figure 1:**
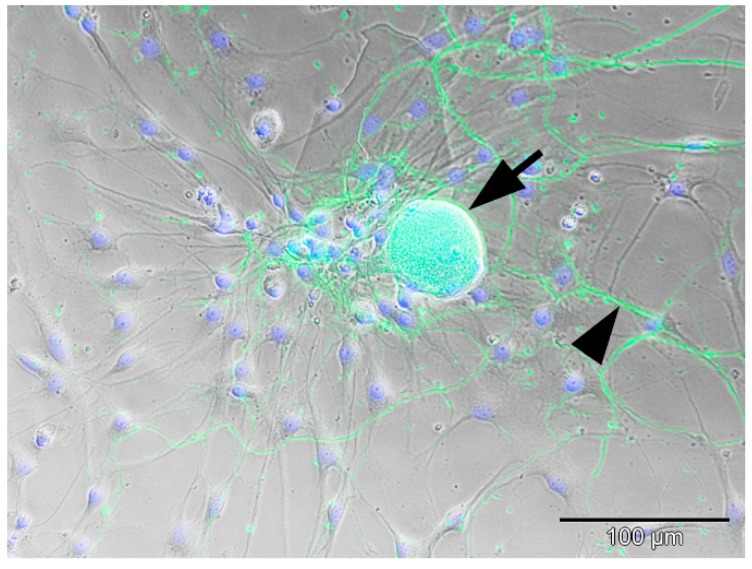
Overlay of phase-contrast and immunofluorescence-images of a non-infected, non-cryopreserved canine DRG neuron at 6 DiV: green = pan-NF; blue = bisBenzimide nuclear stain. The neuron has a large, granular cell body with a phase-bright halo (arrow), is adherent to the substrate, and shows outgrowth of branched neurites (arrowhead). It is surrounded by spindeloid, bi- to multipolar glial cells with delicate processes (blue nuclei) lacking neurofilament staining. Pan-NF = pan-neurofilament, DiV = days in vitro, DRG = dorsal root ganglion.

**Figure 2:**
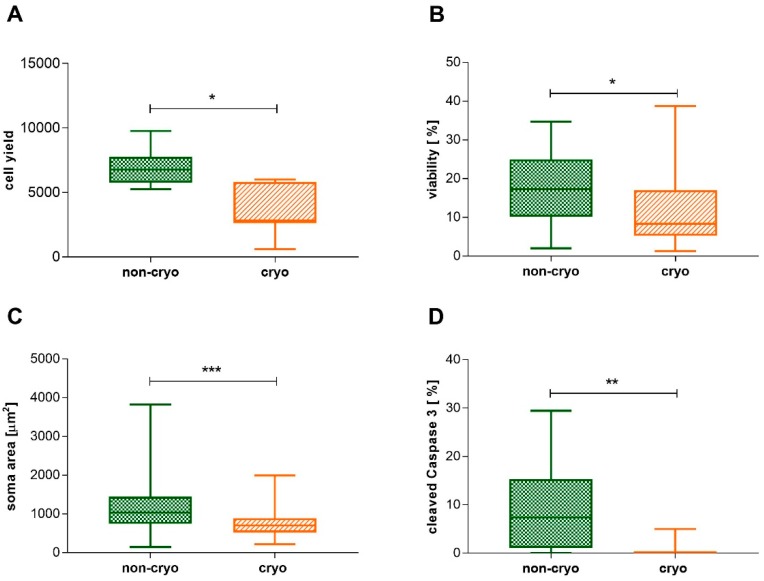
**A:** Y axis: neuronal cell yield [number of neurons] in cell suspensions after isolation/thawing, respectively. Cryo cultures show reduced neuronal cell yield when compared to non-cryo cultures. Non-cryo and cryo: *n* = 8 dogs. **B:** Y axis: neuronal viability [%] at 6 DiV. Cryo neurons show reduced viability when compared to non-cryo neurons. Non-cryo and cryo: *n* = 24 wells. **C:** Y axis: area of neuronal soma [µm^2^] at 6 DiV. The cell bodies of cryo neurons are generally smaller than the ones of non-cryo neurons. Non-cryo: *n* = 98 neurons, cryo: *n* = 70 neurons. **D:** Y axis: neurons expressing cleaved Caspase 3 [%]. A higher percentage of non-cryo neurons express the apoptotic marker cleaved Caspase 3 when compared to cryo neurons. Non-cryo and cryo: *n* = 12 wells. * = *p* < 0.05, ** = *p* < 0.01, *** = *p* < 0.001. Employed statistical test: Mann-Whitney U-test. DiV = days in vitro, non-cryo = non-cryopreserved, cryo = cryopreserved. Graphs show median value, quartils, minimum and maximum values.

Neurons within non-cryopreserved (non-cryo) and cryopreserved (cryo) cultures were adherent to the culture dish and had large, granular cell-bodies with a phase-bright halo (Figure 1). Phase contrast microscopical observation at 24 h in culture revealed the presence of neurons showing outgrowth of multiple branched neurites within bot non-cryo and cryo cultures. Accompanying glial cells were spindeloid, mostly bi- and sometimes multipolar, and had extended delicate processes (Figure 1). Overall, there was a significant reduction in neuronal cell-yield (*p* < 0.05; Mann-Whitney U-test) and viability (*p* < 0.05; Mann-Whitney U-test) in the cryo group (Figure 2A,B). Neuronal cell yield in cell-suspensions of non-cryo cultures averaged 7000 neurons per dog (+/− 1507.07), while cryo cultures yielded a mean of 3457 neurons per dog (+/− 1920.81). Therefore, a mean of 46.67% (+/− 19.7) of neurons were still present in cell suspensions after thawing. The average number of neurons present within each well at 6 days in vitro (DiV) was also significantly higher (*p* < 0.05; Mann-Whitney U-test) in non-cryo than in cryo cultures (Table 1), accounting for a mean neuronal viability of 17.31% (+/− 9.1) in non-cryo and 12.03% (+/− 10.4) in cryo cultures. The absolute number of non-neuronal cells (glial cells) was additionally significantly reduced (*p* < 0.001; Mann-Whitney U-test) in cryo cultures (Table 1). Nevertheless, the proportion of neurons and glial cells and thus the overall-composition of the cultures was not significantly altered (Table 1). The mean soma area of non-cryo neurons (1175.52 µm^2^ +/− 646.12) was significantly larger (*p* < 0.001; Mann-Whitney U-test) than that of cryo neurons (793.6 µm^2^ +/− 399.47; Figure 2C). The percentage of neurons with a diameter larger than 40 µm (referred to in literature as type A neurons; [54]) was significantly decreased following cryopreservation (*p* < 0.001; Fisher’s exact test) from 34.7% in non-cryo cultures to 11.43% in cryo cultures. A significantly higher (*p* < 0.01; Mann-Whitney U-test) percentage of non-cryo neurons (9.11% +/− 8.85) expressed the apoptotic marker cleaved Caspase 3 when compared to cryo neurons (0.68% +/− 1.63) (Figure 2D).

### 2.2. Non-Infected Cultures, Non-Cryopreserved and Cryopreserved: Neurite-Outgrowth

**Figure 3:**
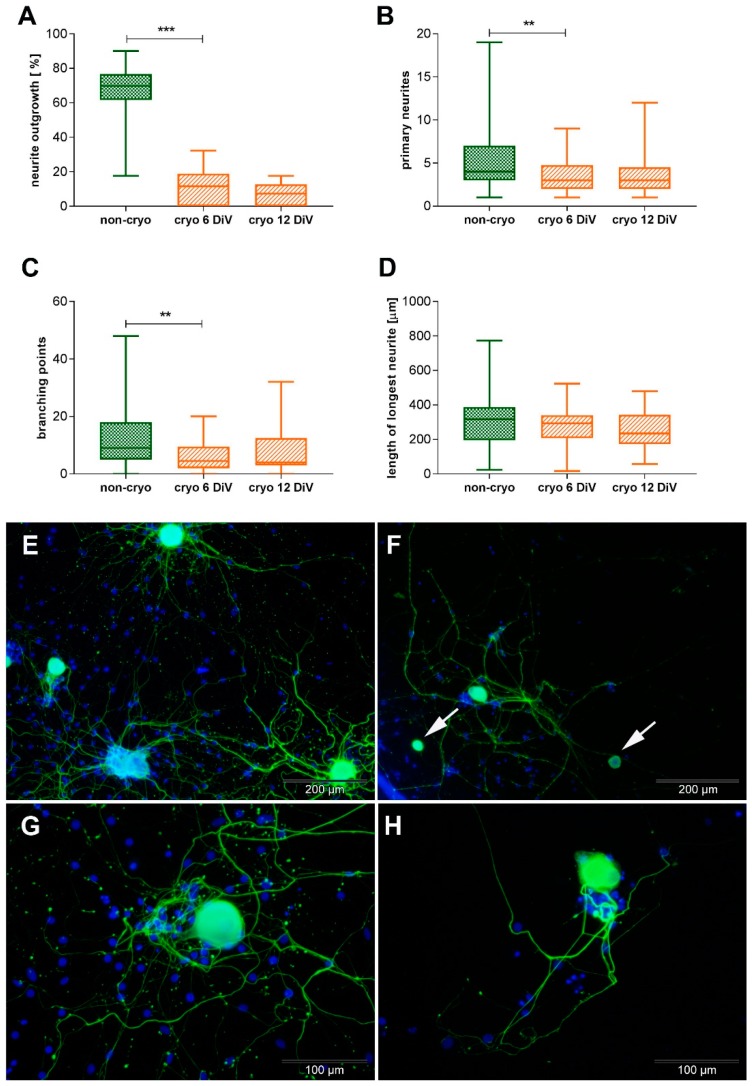
Comparative morphometric analysis of non-infected non-cryo (6 DiV) and cryo (6 and 12 DiV) neurons. **A:** Y axis: neurons showing neurite outgrowth [%]. Non-cryo cultures contain a higher percentage of neurons showing neurite outgrowth compared to cryo cultures. Non-cryo 6 DiV, cryo 6 DiV, and cryo 12 DiV: *n* = 24 wells. **B:** Y axis: number of primary neurites per neuron. Among the neurons showing neurite outgrowth, non-cryo neurons possess a higher number of primary neurites per neuron. Non-cryo: *n* = 72 neurons, cryo 6 DiV: *n* = 24 neurons, cryo 12 DiV: *n* = 37 neurons. **C:** Y axis: number of neurite branching points per neuron. Non-cryo neurons form a higher number of neurite branching points per neuron than cryo neurons. Non-cryo: *n* = 72 neurons, cryo 6 DiV: *n* = 24 neurons, cryo 12 DiV: *n* = 37 neurons. **D:** Y axis: length of longest neurite [µm] per neuron. Non-cryo and cryo neurons show no significant difference in the mean length of their longest neurite. Non-cryo: *n* = 72 neurons, cryo 6 DiV: *n* = 24 neurons, cryo 12 DiV: *n* = 37 neurons. **E–H:** Immunofluorescence-images of non-cryo (**E,G**) and cryo neurons (**F,H**) at 6 DiV; green = pan-NF, blue = bisBenzimide nuclear stain. More neurons without neurites (**F:** arrows) can be found in cryo cultures. Cryo neurons generally show fewer and less branched neurites and smaller cell bodies (**F,H**) compared to non-cryo neurons (**E,G**). ** = *p* < 0.01, *** = *p* < 0.001. Employed statistical test: Mann–Whitney U-test. Pan-NF = pan-neurofilament, DiV = days in vitro, non-cryo = non-cryopreserved, cryo = cryopreserved. Graphs show median value, quartils, minimum and maximum values.

At 6 DiV, a mean of 67.42% (+/− 15.58) of neurons within the non-cryo cultures showed neurite-outgrowth, while the percentage of neurons showing neurite outgrowth was significantly reduced in the cryo cultures to a mean of 10.82% (+/− 10.73; *p* < 0.001; Mann-Whitney U-test; Figure 3A,E–H). Within the population of neurons showing neurite-outgrowth, the number of primary neurites per neuron at 6 DiV was significantly higher in non-cryo cultures (mean: 5.88 primary neurites/ neuron +/− 4.39) compared to cryo cultures (mean: 3.17 primary neurites/ neuron +/− 2.01; *p* < 0.01; Mann-Whitney U-test; Figure 3B,E–H). The average number of neurite-branching points per neuron in non-cryo cultures was 11.78 branching points/ neuron (+/− 9.94), whereas in cryo cultures it was significantly reduced to 6.58 branching points/ neuron (+/− 5.68; *p* < 0.01; Mann-Whitney U-test; Figure 3C,E–H). There was no significant difference in the length of the longest neurite per neuron, which measured a mean 302.97 µm (+/− 135.22) in non-cryo and 275.42 µm (+/− 126.99) in cryo neurons (Figure 3D). Due to the significant differences regarding neurite outgrowth and neurite branching observed in cryo compared to non-cryo neurons, morphometric analysis was additionally performed at 12 DiV for cryo neurons in order to find out whether their neurite ourgrowth improves over time in culture. This was not the case: when comparing cryo cultures at 6 and 12 DiV, there was no significant difference in any of the investigated parameters (percentage of neurons showing neurite-outgrowth, number of primary neurites, number of branching points, length of longest neurite; Figure 3A–D).

### 2.3. Non-Cryopreserved and Cryopreserved Cultures: Susceptibility to Canine Distemper Virus (CDV) Infection

**Figure 4:**
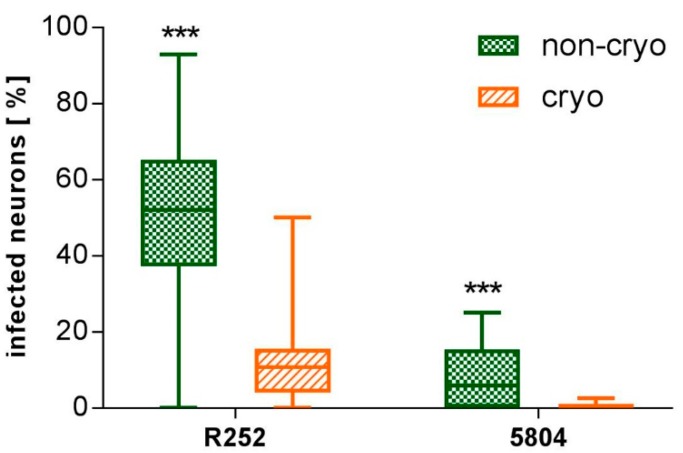
Y axis: neurons infected with different CDV strains [%] at 6 dpi. Cryo neurons show reduced susceptibility to infection with both CDV-strains (CDV R252 and CDV-5804 PeGFP) when compared to non-cryo neurons. Generally CDV R252 infects a higher percentage of neurons than CDV-5804 PeGFP. Non-cryo and cryo: *n* = 24 wells. *** = *p* < 0.001. Employed statistical test: Mann–Whitney U-test., dpi = days post infection, non-cryo = non-cryopreserved, cryo = cryopreserved, CDV = canine distemper virus, eGFP = enhanced green fluorescent protein. Graphs show median value, quartils, minimum and maximum values.

**Figure 5:**
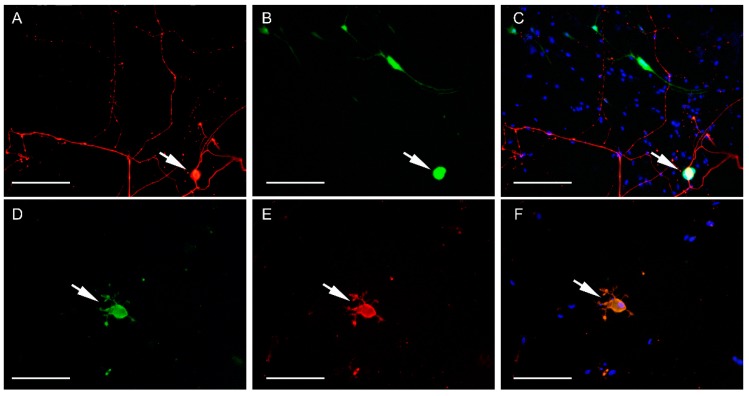
Immunofluorescence images of non-cryo neurons infected with CDV-5804 PeGFP (**A–C**) and CDV R252 (**D–F**) at 6 dpi. **A:** NF (red) indicates neuron with several branched neurites (arrow). **B:** eGFP-expression (green) indicating infection by CDV -5804 PeGFP in a neuron (arrow) and several glial cells. **C:** Merged image of A and B including bisBenzimide nuclear stain (blue) shows expression of eGFP indicating infection with CDV-5804 PeGFP in the cell body of the neuron (arrow) and in the cell bodies and processes of glial cells. **D:** NF (green) indicating neuron (arrow) with short, branched neurites. **E:** Red signal (CDV-nucleoprotein) is visible in the cell-body as well as the neurites of a non-cryopreserved neuron (arrow). **F:** Merged image of D and E including bisBenzimide nuclear stain (blue) indicating a neuron infected with CDV R242 (arrow) with CDV nucleoprotein present in the cell body and neurites. **A–C:** Bar = 200 µm. **D–F:** Bar = 100 µm. Non-cryo = non-cryopreserved, CDV = canine distemper virus, eGFP = enhanced green fluorescent protein, dpi = days post-infection, NF = neurofilament.

CDV-5804 PeGFP-infected neurons were directly traceable via eGFP-expression in living neuronal cultures. They were observed in non-cryo and cryo cultures as soon as 2 dpi. Quantitative evaluations of the percentage of neurons infected by both virus strains (CDV-5804 PeGFP and CDV R252) were carried out at 6 days post-infection (dpi). At this point, a significantly higher percentage of neurons (*p* < 0.001; Mann-Whitney U-test) in both non-cryo and cryo cultures were infected by CDV R252 (non-cryo mean: 49.32% +/− 23.19; cryo mean: 11.5 +/− 10.9) compared to CDV-5804 PeGFP (non-cryo mean: 7.89% +/− 8.21; cryo mean: 0.47% +/− 0.88; Figure 4). A significantly higher percentage of neurons (*p* < 0.001; Mann-Whitney U-test) was infected by both virus strains in non-cryo compared to cryo cultures (Figure 4). Absolute numbers of infected neurons in non-cryo cultures ranged from 0–32 infected neurons per well in CDV R252-infected cultures and 0–5 infected neurons per well in CDV-5804 PeGFP-infected cultures. Cryo cultures showed 0–11 infected neurons per well in the case of CDV R252 and 0–3 infected neurons per well in the case of CDV-5804 PeGFP. While CDV R252-infected neurons were observed in non-cryo and cryo cultures for all 8 dogs investigated, CDV-5804 PeGFP-infected neurons were not observed in 1 out of 8 dogs in non-cryo and in 4 out of 8 dogs in cryo cultures. For both investigated virus strains, viral antigen was observed in the cell body of neurons (Figure 5). In addition CDV R252 viral protein was rarely observed within neurites of infected neurons (Figure 5). Neurons infected with either virus strain did not show any morphological alterations visible in phase contrast microscopy. 

## 3. Discussion

The present study aimed to establish canine DRG neurons as a promising in vitro model system to study the neuropathogenesis of neurotropic virus infections with a non-rodent host spectrum, such as CDV, a disease affecting mainly carnivores. Furthermore, the possibility of cryopreserving canine DRG neurons and its effect upon their infectability with CDV were tested in order to make this in vitro system more accessible for a large scale routine use in cell culture. 

The results revealed that morphologically intact neurons showing neurite outgrowth can be recovered after cryopreservation of dissociated canine DRG neurons. The percentage of neurons within the cultures was not altered, when compared with non-cryo cultures, and the cryo neurons were shown to be able to remain in culture for at least 12 days. Neurite-bearing neurons were observed within both non-cryo and cryo cultures at 24 h in culture. Nonetheless, the absolute number of neurons (cell yield) as well as the viability, the percentage of neurons showing neurite-outgrowth, the number of primary neurites per neuron, the neurite-branching and the susceptibility to infection with CDV were significantly reduced in cryo compared to non-cryo neurons. Those results indicate that cryo canine DRG neurons represent a practicable alternative to non-cryo neurons, but that they are not completely representative of their non-cryo counterpart. This observation is in contrast to numerous studies upon the cryopreservation of fetal [33,34] and neonatal [41,44] rodent neurons. 

A standard cryopreservation procedure was selected in the present study, which has previously been successfully applied to postnatal rat DRG-neurons [44] and includes freezing of the dissociated neurons in a cryopreservation medium with a DMSO-content of 10%, dropping of the temperature at a rate of 1 °C per minute, and rapidly thawing the neurons in a water bath at 37 °C. In general, cryopreservation of cells results in a loss of cell yield and viability [51], which was an expected observation in the current study. Cryo canine DRG neurons showed a viability of 12.03% at 6 DiV, which is lower than in studies investigating cryopreservation of neonatal rat neurons ([43]: 28.6 +/− 2.7%) or neurons from rat fetuses ([33]: 90.2%). This observation can be explained by the fact that mature (adult) neurons are more vulnerable to experimental processing than immature (fetal) neurons [39] in general and thus yield less viable cells to start with. Furthermore, in most of the reviewed studies the viability of the neurons was determined in dissociated cell suspensions directly after isolation or thawing, respectively [33,39], not taking into account that there is often great neuronal loss during the first 24 h in culture [50]. In the present study, on the other hand, the viability was assessed on the basis of the ability of neurons to survive in culture until 6 DiV, therefore including the loss of neurons taking place during the first days in culture. This might additionally account for part of the relatively low viability observed in comparison to other studies. 

The loss of cells in the present study was not limited to neurons but also affected glial cells, leading to an overall unchanged proportion of neurons and glial cells as similarly observed in studies using human fetuses [49] and rat fetuses [33]. This is in contrast to a study using bovine fetuses [43]. A low percentage of neurons within both cryo and non-cryo cultures, as in the present case, has also been observed in a study using cerebral neurons of rat fetuses [33] and can be attributed to the fact that glial cells proliferate, while neurons are in a post-mitotic state. The fact that the investigated cultures consist of a mixture of both neurons and DRG glial cells represents one limitation of the present study. The presence of non-neuronal cells certainly has an influence upon neuronal viability and functional characteristics and thus the establishment of pure glial cell-free canine DRG cultures could represent a promising goal for future studies upon the effects of neuronal CDV infection. However, cultures consisting of both neurons and glial cells also provide a model that more closely mimics the natural environment in order to study the pathogenesis of CDV infection and thus there is a strong legitimation for neuroinfection research on such mixed cultures.

Regarding the morphology of non-cryo and cryo neurons, cells with a typical neuronal morphology and attachment to the culture dish were observed in both non-cryo and cryo cultures in the present study. Nevertheless, when considering neurite outgrowth as a functional readout, there was a significant decline in cryo compared to non-cryo cultures regarding the percentage of neurons showing neurite outgrowth, number of primary neurites and branching points formed. This might seem surprising, considering the fact that there are numerous studies stating that non-cryo and cryo neurons from different species are morphologically indifferent [33,35,38,41,45,48,50]. But at a second glance, most of those studies are lacking an objective quantification of neurite outgrowth and branching points. Only a few studies have investigated this in detail [34,44], indicating that the change in those parameters might have been neglected before in cryo neurons. One of the reasons for the decreased neurite outgrowth observed in the cryo neurons might be the fact that, even though relative proportions of neurons and glial cells were unchanged, there was an absolute decline of glial cell numbers within the cryo cultures. In fact, the phenotype of glial cells (satellite glial cells) within canine DRG has been recently characterized in detail in situ and in vitro [32]. The latter study revealed the presence of a unique glial cell population with characteristics of astrocytes, oligodendrocytes, and precursor cells as well as neurite outgrowth-promoting effects. At 2 DiV the majority of non-neuronal cells within non-cryo canine DRG neuronal cultures express GFAP (glial fibrillary acidic protein; median: 85%; min.–max.: 84–92%), CNPase (2’,3’-cyclic-nucleotide 3’-phosphodiesterase; median. 78%; min.–max.: 77–83%) and Vimentin (median: 82%; min.–max.: 77–84%), whereas only 11% (min.–max.: 9–19%) of them express S100. Interestingly, the majority of the glial cells co-express GFAP and CNPase (median: 72%; min.–max.: 71–75%), which are typical markers for astrocytes and oligodendrocytes, respectively (unpublished data, generated from DRG neuronal cultures of 5 healthy beagle dogs). Different types of canine glial cells have further been shown to promote neurite-outgrowth in a human neuronal cell line (hNT2; [55]). Therefore, the reduced density of glial cells within the wells of cryo canine DRG neurons may indeed represent one factor that explains the decreased neurite outgrowth, observed in the present study. Consequently, there is a need for further studies investigating whether the co-culture of cryo canine DRG neurons with canine satellite glial cells can improve their neurite outgrowth.

The present study revealed that the average length of the longest neurite was unchanged in cryo compared to non-cryo neurons, indicating that those neurites present were not impaired in terms of their growth capacity. Another interesting observation was the reduction of soma size and percentage of neurons measuring more than 40 µm in diameter following cryopreservation. DRG neurons can be subdivided according to their size into larger neurons (bright cytoplasm, 30–75 µm in diameter, previously referred to as type A neurons) and smaller neurons (dark cytoplasm, 15–40 µm in diameter, previously referred to as type B neurons; [54]). The reduction of soma size and diameter of cryo neurons could indicate a more pronounced effect of cryopreservation upon DRG sensory neurons with a diameter larger than 40 µm. Recent studies using large-scale single-cell RNA sequencing have revealed 11 functionally different types of sensory neurons within the DRG of mice with marked differences in molecular characteristics and the stimuli they react to [56]. An immunohistological study of canine DRG neurons in situ revealed that around 20% of the sensory neurons expressed calcitonin gene-related peptide (CGRP), which is a marker for peptidergic neurons [57]. It was expressed in small to medium-sized neurons, which is in accordance with in vitro observations on murine DRG neurons [58]. On the other hand, isolectin B4-positive, non-peptidergic neurons have been detected independent of soma size in mice [58], indicating that no conclusions upon the functional subtype of sensory neurons can be drawn from the soma-size alone. Previous data revealed that the majority (median: 70%, min.–max.: 67–73%; unpublished data generated from DRG neuronal cultures of 5 healthy beagle dogs) of canine DRG neurons with a diameter larger than 40 µm express the p75 neurotrophin receptor (p75^NTR^) in vitro at 2 DiV, which is a receptor for nerve growth factor (NGF). Since the division of DRG neurons into subtypes according to their diameter represents a merely morphological classification, further investigations on the impact of cryopreservation upon different functional subtypes of sensory neurons, namely peptidergic and non-peptidergic neurons, will be needed in future.

Since apoptosis as opposed to necrosis was found to be the major mechanism of cell death during cryopreservation [59], the expression of cleaved caspase 3 was comparatively investigated in non-cryo and cryo neurons at 6 DiV. Cleaved caspase 3 is an executioner caspase playing an important role in the final phase of apoptosis. In the present study, there was no increased expression of the apoptotic marker in cryo neurons. Surprisingly, the contrary was the case: The expression of the apoptotic marker was significantly reduced in cryo compared to non-cryo neurons. This indicates that, at least at 6 DiV, apoptosis does not seem to be a major issue of cell death in cryo canine DRG neurons. On the other hand, cryo neurons that underwent apoptosis due to freezing and thawing might simply not be detectable anymore in cryo cultures at 6 DiV due to lack of attachment to the culture plates of dead or dying neurons [60,61]. Whether the success of cryopreservation of canine primary DRG neurons can be enhanced by caspase-inhibition prior to freezing, as observed in human embryonic stem cell-derived neurons [39], remains to be investigated. 

Regarding CDV infection and irrespective of cryopreservation, a higher susceptibility of primary canine DRG neurons to infection with the neurotropic, CDV strain R252 was observed in comparison to the mustelid strain CDV-5804 PeGFP. This observation is not surprising, considering the fact that CDV-5804 PeGFP [20] is a virus strain originally isolated from ferrets, while CDV R252 [18] is a neurotropic strain isolated from an infected dog and, therefore, more prone to infect canine neurons than CDV-5804 PeGFP. Similar results have also been obtained in in vitro studies infecting canine schwann cells, schwann cell-like brain glia, fibroblasts and olfactory ensheathing cells [17,62]. Regarding the comparative infection of non-cryo and cryo canine DRG neurons, a reduced susceptibility to CDV infection has been observed in cryo neurons. This is in contrast to a previous publication comparatively investigating the infection by GFP-expressing lentiviruses of cortical neurons derived from mouse embryos, where a similar infection efficiency was observed in non-cryo and cryo neurons [34]. The reduced infectability of the cryo DRG neurons with CDV observed in the present study is possibly due to alterations in certain membrane-bound molecules provoked by cryopreservation. Since the receptor on the cell membrane of neurons that CDV uses to enter those cells is unidentified so far, further investigations on this will be needed in the future. The very low absolute numbers of non-cryo and cryo neurons infected by CDV-5804 PeGFP observed in the current study and the fact that not all of the investigated 8 dogs provided DRG neurons (both non-cryo and cryo) that were infectable with this virus strain considerably hamper further investigations upon the impact of CDV infection upon neuronal morphology, neurite outgrowth, branching of neurites and other parameters. Thus, the data indicate that this particular CDV strain might not be as suitable as CDV R252 to study CDV neuronal infection in vitro. Nevertheless, it is an interesting and worth mentioning observation that not only mustelid, but also canine neurons are in general susceptible to infection by CDV-5804 PeGFP.

In conclusion, the use of cryo canine DRG neurons represents a possible alternative to non-cryo neurons, especially considering the better planning of experiments, the possibility to conduct multiple experiments with neurons from one animal, the irregular availability of post-mortem tissue from adult dogs for tissue sampling, and in order to fulfill the aim of reducing and refining animal experiments. Regardless, canine DRG neurons derived from cryo cultures should be thoroughly investigated regarding their morphology and only morphologically intact neurons showing neurite outgrowth should be used for further experiments. Special care should be taken when interpreting results of studies involving virus infection with cryo neurons, particularly regarding the percentage of infected neurons, as this cannot be extrapolated directly to non-cryo neurons. For future investigations upon CDV neuronal infection, CDV R252 should be preferably used as opposed to CDV-5804 PeGFP, as it provides higher absolute numbers of CDV-infected neurons to allow for further investigations. The present study represents the first report of in vitro CDV infection of canine DRG neurons, and its usefulness as an in vitro model for CDV neuronal infection, which is essential for future pathomechanistical investigations. Furthermore, it provides the basis for further studies identifying the so far unknown neuronal receptor for CDV.

## 4. Materials and Methods

### 4.1. Isolation and Cultivation of Primary Canine Dorsal Root Ganglion Neurons

**Figure 6:**
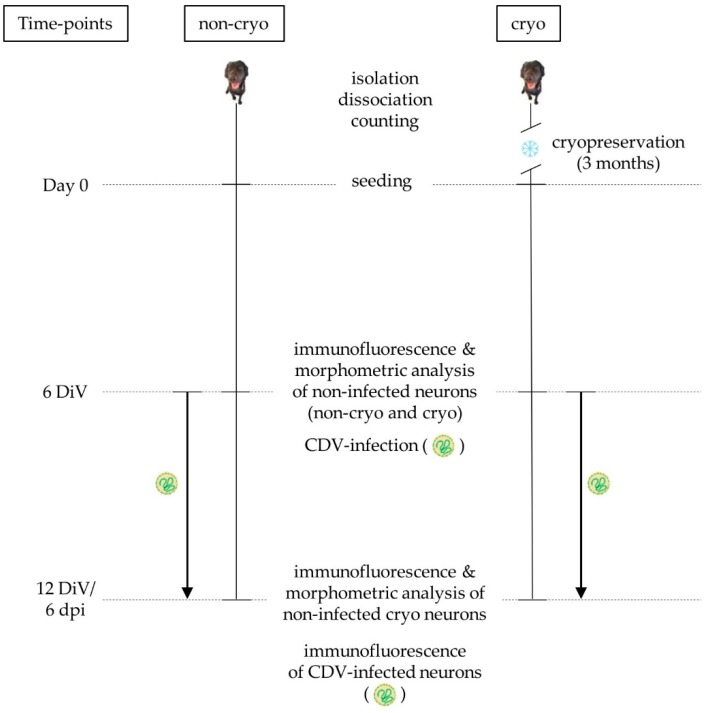
Experimental design: while non-cryo neurons were directly seeded after isolation, dissociation and counting, cryo-neurons were cryopreserved an thawed after 3 months. The day the neurons were seeded represents day 0 of the experiment. Part of the cultures were fixed for comparative immunofluorescence and morphometric analysis at 6 DiV for non-cryo and cryo neurons and at an additional time-point (12 DiV) for cryo neurons. The remaining DRG neuronal cultures were infected with 2 different strains of CDV (CDV-5804 PeGFP, CDV R252) at 6 DiV and kept in culture until 12 DiV or 6 dpi, respectively, when they were processed for immunostaining and further analysis. Non-cryo = non-cryopreserved neurons, cryo = cryopreserved neurons, DiV = days in vitro, dpi = days post infection, CDV = canine distemper virus, eGFP = enhanced green fluorescent protein.

Dorsal root ganglia (DRG) were obtained from 3 female and 5 male, 6 months old, healthy Beagle dogs, which were control animals from an unrelated study (33.19-42502-05-16A043). No animals were killed for the purpose of this particular study. The experiments were approved by the Lower Saxony State Office for Consumer Protection and Food Safety (permission number: 16A 024). Isolation and dissociation of primary DRG neurons was performed as described previously [31] with slight modifications. Briefly, surrounding fat and connective tissue as well as blood vessels were removed from the DRG and they were minced and kept on ice in Hanks’s balanced salt solution (HBSS) 1x (Gibco, Life Technologies; Paisley, UK). Consequently, they were digested with type IV and type XI collagenase, type IV hyaluronidase and trypsin (Sigma-Aldrich Chemie GmbH; Taufkirchen, Germany) for 60 min at 37 °C. Afterwards, they were dissociated using successively narrowed, flame-constricted Pasteur pipettes and the addition of DNase I (Roche Diagnostics GmbH; Mannheim; Germany). Cells were centrifuged and resuspended in Dulbecco’s modified Eagle’s Medium (DMEM; Gibco, Life Technologies; Paisley, UK) supplemented with 10% fetal calf serum (FCS; PAA Laboratories GmbH; Pasching, Austria) and neurons were separated from the other cells using a two-step Percoll-gradient (25% and 27%) (GE HealthCare BioSciences, Fisher Scientific; Rockford, Tempe, AZ, USA). Neurons were resuspended in Sato’s medium [63], counted in a Neubauer chamber (Roth C. GmbH & CO KG; Karlsruhe, Germany), and plated at a density of 150 neurons per well in half-area 96 well microtiter plates (Nunc GmbH & CO KG; Wiesbaden, Germany) coated with poly-L-lysin (100 µg/mL; Sigma-Aldrich Chemie GmbH; Taufkirchen, Germany) and laminin (Becton, Dickinson and Company; Franklin Lakes, NJ, USA). Neurons were identified in the Neubauer chamber by their large, round, granular cell-body, which was surrounded by a phase-bright halo [46]. Neurons were maintained in Sato’s medium supplemented with 1% bovine serum albumin (BSA; Sigma-Aldrich Chemie GmbH; Taufkirchen, Germany) and 2.1 ng/100 µL nerve growth factor (NGF; Peprotec Inc., Tebu-Bio; Frankfurt, Germany). Cultures were kept in an incubator at 37 °C and 5% CO_2_. Twice weekly 60% of the medium was exchanged with fresh medium. Non-cryo and cryo neurons were observed by phase contrast microscopy at 24 h in culture in order to study their morphology and assess the general presence of neurite outgrowth. Quantification of neurite outgrowth was carried out after fixation and immunofluorescence at 6 DiV (Figure 6).

### 4.2. Cryopreservation and Cell Yield

Cryopreservation of DRG neurons was performed according to a previously published protocol with slight modifications [44]. After dissociation, half of the cells obtained from each animal were directly seeded, and the other half was resuspended in 1 mL freezing medium containing 10% dimethylsulfoxid (DMSO; Sigma-Aldrich Chemie GmbH; Steinheim, Germany) as cryoprotective agent, 31.5% FCS, 0.65% penicillin-streptomycin (PS, PAA Laboratories GmbH; Pasching, Austria) and 57.85% minimum essential medium with Earle’s salts (MEM Earle’s; Biochrom GmbH; Berlin, Germany). Cells were transferred into cryo vials (Thermo Fisher Scientific; Roskilde, Denmark), placed into a freezing container (Thermo Scientific Nalgene; Waltham, MA, USA) and cooled down to −80 °C at a cooling rate of −1 °C/minute. After 24 h, they were placed into liquid nitrogen (−196 °C). After 3 months, cells were thawed at 37 °C and the content of each cryo vial was transferred into 5 mL Sato’s medium (37 °C). In order to remove the cryoprotectant, cells were centrifuged and the pellets were resuspended in 1 mL Sato’s medium supplemented with 1% BSA and 2.1 ng/100 µL NGF. Neurons were counted in a Neubauer-chamber and the neuronal cell yield (total number of neurons present in the cell suspension) was calculated for each dog (*n* = 8). Thereafter, cells were seeded analogous to the non-cryopreserved cells.

### 4.3. Virus Strains and Virus Infection

For virus infection of canine DRG neurons, 2 different strains of CDV were used: The mustelid strain CDV-5804 PeGFP, which has been produced from a wild-type strain lethal for ferrets by inserting an enhanced green fluorescent protein (eGFP) (kindly provided by V. von Messling; [20]) and the neurotropic strain CDV R252 (kindly provided by S. Krakowka, Ohio State University, Columbus, OH, USA), which was originally isolated from the spleen homogenate of a naturally infected dog [16,18]. Non-cryopreserved and cryopreserved cultures were infected with CDV at 6 DiV (Figure 6). The culture medium was removed and the cells were washed twice with MEM Earle’s containing 1% PS to remove residual serum-containing culture medium. The virus was added diluted in cold (4 °C) MEM Earle’s containing 1% PS at a multiplicity of infection (MOI) of 1000 virus particles per neuron. Cultures were incubated at 37 °C and 5% CO_2_ for 2–3 h before removing the virus dilution, washing twice with MEM Earle’s containing 1% PS and adding fresh Sato’s medium supplemented with 1% BSA and 2.1 ng/100 µL NGF. All media containing viruses were handled on ice. Non-infected control cultures for each virus were handled the same way, except that they were incubated with cold (4 °C) MEM Earle’s containing 1% PS only, instead of virus dilution. At 3 dpi, 60% of the medium of the cultures was replaced by fresh medium. Cultures were fixed at 6 dpi using paraformaldehyde (4%) and mounted with phosphate-buffered saline (PBS) for further processing.

### 4.4. Immunofluorescence

To identify DRG neurons within the cultures, quantify their neurite outgrowth, detect apoptotic neurons, and evaluate their infection with CDV, the cultures were stained with anti-human neurofilament protein (NF) antibody (2F11; mouse monoclonal; 1:100; Dako GmbH, Jena, Germany), anti-cleaved caspase 3 antibody (Asp175; rabbit polyclonal; 1:900; Cell signaling Technology; Frankfurt am Main, Germany), anti-CDV nucleoprotein antibody (D110; mouse monoclonal; 1:400; kindly provided by Prof. A. Zurbriggen; Vetsuisse faculty, University of Bern, Bern, Switzerland), and a directly fluorescent antibody against the heavy neurofilament protein (NF-H AF488; NF-01; mouse monoclonal; 1:500; Novus Biologicals; Abingdon, UK) in order to double-stain CDV nucleoprotein and neurons in CDV R252-infected cultures. On 6 (cryopreserved and non-cryopreserved) and 12 DiV (cryopreserved; Figure 6), cells were washed with PBS and fixed with paraformaldehyde (4%) for 20 min. For identification of the intracellular antigens, cells were permeabilized with PBS supplemented with 0.25% TritonX (PBST; Merck; Darmstadt, Germany). Unspecific bindings were blocked with goat serum (5%) and cultures were incubated with primary antibodies overnight at 4 °C. Specificity of immunostaining was verified by omitting primary antibodies as well as the presence of cell-specific staining patterns. The next day, cultures were washed with PBST and secondary antibodies were added according to the host species of the primary antibodies at a dilution of 1:200 (Cy^TM^3-conjugated goat anti-rabbit IgG antibody, Cy^TM^3-conjugated goat anti-mouse IgG antibody: Jackson ImmonoResearch Laboratories; Hamburg, Germany; Alexa Fluor 488-conjugated goat-anti-mouse IgM antibody: Invitrogen, Life Technologies; Paisley, UK) and incubated for 2 h at room temperature. Afterwards, nuclear staining of the cultures was performed with bisBenzimide (Sigma-Aldrich Chemie GmbH; Taufkirchen, Germany). For further investigations, cultures were mounted with PBS.

### 4.5. Comparative Analysis of Cell Morphology, Viability, Cleaved Caspase 3-Expression and Neurite Outgrowth of Non-Infected, Non-Cryopreserved and Cryopreserved Neurons

Phase contrast and fluorescence microscopy of the cultures were performed using an inverted fluorescence microscope (Olympus IX70-S8F2; Olympus Life Science Europe GmbH; Hamburg, Germany) and a reflected fluorescence system (Olympus U-RFL-T; Olympus Life Science Europe GmbH; Hamburg, Germany). Photomicrographs were taken using the Olympus Microscope Digital Camera DP72 as well as the imaging software cell^F (Olympus Soft Imaging Solutions GmbH; Münster, Germany). The morphology of the neurons was observed using phase contrast microscopy while in culture and fluorescence microscopic images were acquired after fixation and immunolabelling at DiV 6 and 12, respectively (Figure 6). Viable neurons were defined as cells, which were (i) still present and adherent to the substrate of the culture dish at 6 DiV, (ii) showed a morphologically intact, round cell-body with a phase-bright halo and a clearly visible, round nucleus and (iii) expressed NF [43,46,49,60]. Neuronal viability was calculated as the percentage of neurons originally seeded that were still viable at 6 DiV. To determine the percentage of neurons and non-neuronal cells (glial cells) within the cultures, photomicrographs from 10 randomly selected visual fields per well in a 200× magnification were taken for each non-cryo and cryo neurons at 6 DiV. Using bisBenzimide nuclear staining, the number of NF-expressing cells and the total number of cells was counted and the percentage of NF-positive cells was calculated for each well (*n* = 24 for each non-cryo and cryo). For evaluation of cleaved Caspase 3-expression, photomicrographs of the neurons within 12 wells were taken for each condition (non-cryo and cryo) at a magnification of 200×. Neurons double-labelled for NF and cleaved Caspase 3 were counted, and the percentage of neurons expressing cleaved Caspase 3 at 6 DiV was calculated for each well. In total, 242 non-cryo and 238 cryo neurons were evaluated. For determination of the percentage of neurons showing neurite outgrowth, all neurons stained for NF within each well under each condition were counted at 6 DiV (non-cryo and cryo) and 12 DiV (cryo), and the percentage of neurons which showed outgrowth of at least 1 neurite was determined for each well (*n* = 24 wells for each non-cryo, cryo 6 DiV and cryo 12 DiV). In total, 623 neurons were counted for non-cryo, 433 neurons for cryo 6 DiV and 471 for cryo 12 DiV. Morphometric analysis was performed using the Imaging software analySIS 3.1 (Olympus Soft Imaging Solutions GmbH; Münster, Germany) according to a previously published protocol with slight modifications [64]. For determination of soma area, number of primary neurites, branching points, and length of longest neurite, photomicrographs from 10 randomly selected visual fields per well in a 200× magnification were taken for each condition. The soma area of all the neurons present within these visual fields at 6 DiV was measured and their diameter was determined (non-cryo: *n* = 98 neurons; cryo: *n* = 70 neurons). Neurons were subdivided according to their diameter into neurons with a diameter larger than 40 µm (referred to in literature as type A neurons; [54]) and a diameter smaller than or equal to 40 µm (referred to in literature as type B neurons; [54]). The percentage of type A and type B neurons was calculated in non-cryo and cryo cultures, respectively. For the analysis of number of primary neurites (neurites directly emanating from the soma), neurite branching points, and length of longest neurite, only those neurons showing outgrowth of at least 1 neurite were taken into account (non-cryo: 72 neurons; cryo 6 DiV: 24 neurons; cryo 12 DiV: 37 neurons).

### 4.6. Evaluation of Neuronal CDV-Infection

For evaluation of cytopathogenic effects (CPE), characterized by morphological alterations of neurons, cultures were investigated using an inverted microscope. Cultures infected with the fluorescent virus CDV-5804 PeGFP were additionally observed using an inverted fluorescence microscope on a daily basis in order to detect the earliest time-point when infection of neurons by this virus can be detected. At 6 dpi (Figure 6), cultures infected by both virus strains (CDV-5804 PeGFP and CDV R252) were fixed with paraformaldehyde (4%) and processed for immunofluorescent staining. For each virus strain, total number of neurons and number of infected neurons within each well was counted (*n* = 24 wells for each virus, non-cryo and cryo) and the percentage of infected neurons within each well was calculated. Cells double-labelled for NF and CDV-nucleoprotein or eGFP, respectively, were considered infected neurons.

### 4.7. Illustrations and Graphs

Photomicrographs for illustrations were adjusted regarding contrast, brightness and sharpness, if necessary and figures were arranged using Adobe^®^Photoshop^®^ CS5 (Adobe Systems Inc., San Jose, CA, USA). Graphs were generated using GraphPad Prism 7 (GraphPad Software; La Jolla, CA, USA).

### 4.8. Statistics

Statistical analysis was performed using SPSS^®^ 25 for windows (SPSS Inc., Chicago, IL, USA). First, an explorative data analysis was undertaken and a Shapiro-Wilk test of normality was employed. Since not all of the data were normally distributed, non-parametric tests (Mann-Whitney U-test; Fisher’s exact test) were used for statistical analysis. Statistical significance was designated as *p* < 0.05.

## Figures and Tables

**Table 1:** Cellular composition of non-cryo and cryo cultures at 6 DiV.

Condition	Non-Cryo	Cryo
glial cells [number/well]	470 *** (+/− 164.21)	225 *** (+/− 104.65)
glial cells [%/well]	94.63 (+/− 2.56)	93.20 (+/− 4.02)
neurons [number/well]	26 * (+/− 13.66)	18 * (+/−15.6)
neurons [%/well]	5.37 (+/− 2.56)	6.80 (+/− 5.66)

Values represent mean +/− SD; *n* = 24 wells (8 different dogs, 3 wells per dog and condition); Non cryo = non-cryopreserved, cryo = cryopreserved, DiV = days in vitro, SD = standard deviation. * = *p* < 0.05, *** = *p* < 0.001. Employed statistical test: Mann-Whitney U-test.

## References

[1] Buch A., Muller O., Ivanova L., Dohner K., Bialy D., Bosse J.B., Pohlmann A., Binz A., Hegemann M., Nagel C.H. (2017). Inner tegument proteins of Herpes Simplex Virus are sufficient for intracellular capsid motility in neurons but not for axonal targeting. PLoS Pathog..

[2] Gaburro J., Bhatti A., Sundaramoorthy V., Dearnley M., Green D., Nahavandi S., Paradkar P.N., Duchemin J.B. (2018). Zika virus-induced hyper excitation precedes death of mouse primary neuron. Virol. J..

[3] Omar M., Bock P., Kreutzer R., Ziege S., Imbschweiler I., Hansmann F., Peck C.T., Baumgärtner W., Wewetzer K. (2011). Defining the morphological phenotype: 2’,3’-cyclic nucleotide 3’-phosphodiesterase (CNPase) is a novel marker for in situ detection of canine but not rat olfactory ensheathing cells. Cell Tissue Res..

[4] Techangamsuwan S., Imbschweiler I., Kreutzer R., Kreutzer M., Baumgärtner W., Wewetzer K. (2008). Similar behaviour and primate-like properties of adult canine Schwann cells and olfactory ensheathing cells in long-term culture. Brain Res..

[5] Jeffery N.D., Smith P.M., Lakatos A., Ibanez C., Ito D., Franklin R.J. (2006). Clinical canine spinal cord injury provides an opportunity to examine the issues in translating laboratory techniques into practical therapy. Spinal Cord..

[6] Spitzbarth I., Bock P., Haist V., Stein V.M., Tipold A., Wewetzer K., Baumgärtner W., Beineke A. (2011). Prominent microglial activation in the early proinflammatory immune response in naturally occurring canine spinal cord injury. J. Neuropathol. Exp. Neurol..

[7] Wewetzer K., Radtke C., Kocsis J., Baumgärtner W. (2011). Species-specific control of cellular proliferation and the impact of large animal models for the use of olfactory ensheathing cells and Schwann cells in spinal cord repair. Exp. Neurol..

[8] Beineke A., Puff C., Seehusen F., Baumgärtner W. (2009). Pathogenesis and immunopathology of systemic and nervous canine distemper. Vet. Immunol. Immunopathol..

[9] Griot C., Vandevelde M., Schobesberger M., Zurbriggen A. (2003). Canine distemper, a re-emerging morbillivirus with complex neuropathogenic mechanisms. Anim. Health Res. Rev..

[10] Nesseler A., Baumgärtner W., Gaedke K., Zurbriggen A. (1997). Abundant expression of viral nucleoprotein mRNA and restricted translation of the corresponding viral protein in inclusion body polioencephalitis of canine distemper. J. Comp. Pathol..

[11] Nesseler A., Baumgärtner W., Zurbriggen A., Orvell C. (1999). Restricted virus protein translation in canine distemper virus inclusion body polioencephalitis. Vet. Microbiol..

[12] Liebert U.G., Baczko K., Budka H., ter Meulen V. (1986). Restricted expression of measles virus proteins in brains from cases of subacute sclerosing panencephalitis. J. Gen. Virol..

[13] Isaacson S.H., Asher D.M., Godec M.S., Gibbs C.J., Gajdusek D.C. (1996). Widespread, restricted low-level measles virus infection of brain in a case of subacute sclerosing panencephalitis. Acta Neuropathol..

[14] Noyce R.S., Delpeut S., Richardson C.D. (2013). Dog nectin-4 is an epithelial cell receptor for canine distemper virus that facilitates virus entry and syncytia formation. Virology.

[15] Tatsuo H., Ono N., Yanagi Y. (2001). Morbilliviruses use signaling lymphocyte activation molecules (CD150) as cellular receptors. J. Virol..

[16] Orlando E.A., Imbschweiler I., Gerhauser I., Baumgärtner W., Wewetzer K. (2008). In vitro characterization and preferential infection by canine distemper virus of glial precursors with Schwann cell characteristics from adult canine brain. Neuropathol. Appl. Neurobiol..

[17] Techangamsuwan S., Haas L., Rohn K., Baumgärtner W., Wewetzer K. (2009). Distinct cell tropism of canine distemper virus strains to adult olfactory ensheathing cells and Schwann cells in vitro. Virus Res..

[18] Confer A.W., Kahn D.E., Koestner A., Krakowka S. (1975). Biological properties of a canine distemper virus isolate associated with demyelinating encephalomyelitis. Infect. Immun..

[19] Summers B.A., Greisen H.A., Appel M.J.G. (1984). Canine distemper encephalomyelitis: Variation with virus strain. J. Comp. Pathol..

[20] Von Messling V., Milosevic D., Cattaneo R. (2004). Tropism illuminated: Lymphocyte-based pathways blazed by lethal morbillivirus through the host immune system. Proc. Natl. Acad. Sci. USA.

[21] Luhmann H.J., Hanganu I., Kilb W. (2003). Cellular physiology of the neonatal rat cerebral cortex. Brain Res. Bull..

[22] Lu J., Karadsheh M., Delpire E. (1999). Developmental regulation of the neuronal-specific isoform of K-Cl cotransporter KCC2 in postnatal rat brains. J. Neurobiol..

[23] Wallis K., Sjogren M., van Hogerlinden M., Silberberg G., Fisahn A., Nordstrom K., Larsson L., Westerblad H., de Escobar G.M., Shupliakov O. (2008). Locomotor deficiencies and aberrant development of subtype-specific GABAergic interneurons caused by an unliganded thyroid hormone receptor alpha1. J. Neurosci..

[24] Schock S.C., Jolin-Dahel K.S., Schock P.C., Theiss S., Arbuthnott G.W., Garcia-Munoz M., Staines W.A. (2012). Development of dissociated cryopreserved rat cortical neurons in vitro. J. Neurosci. Methods.

[25] Russell W.M.S.B.R. (1959). The Principles of Humane Experimental Technique.

[26] Amir R., Michaelis M., Devor M. (1999). Membrane potential oscillations in dorsal root ganglion neurons: Role in normal electrogenesis and neuropathic pain. J. Neurosci..

[27] An L., Li G., Si J., Zhang C., Han X., Wang S., Jiang L., Xie K. (2016). Acrylamide Retards the Slow Axonal Transport of Neurofilaments in Rat Cultured Dorsal Root Ganglia Neurons and the Corresponding Mechanisms. Neurochem. Res..

[28] Gold M.S., Dastmalchi S., Levine J.D. (1996). Co-expression of nociceptor properties in dorsal root ganglion neurons from the adult rat in vitro. Neuroscience.

[29] Murali S.S., Napier I.A., Mohammadi S.A., Alewood P.F., Lewis R.J., Christie M.J. (2015). High-voltage-activated calcium current subtypes in mouse DRG neurons adapt in a subpopulation-specific manner after nerve injury. J. Neurophysiol..

[30] Scroggs R.S., Fox A.P. (1992). Calcium current variation between acutely isolated adult rat dorsal root ganglion neurons of different size. J. Physiol..

[31] Gerhauser I., Hahn K., Baumgärtner W., Wewetzer K. (2012). Culturing adult canine sensory neurons to optimise neural repair. Vet. Rec..

[32] Tongtako W., Lehmbecker A., Wang Y., Hahn K., Baumgärtner W., Gerhauser I. (2017). Canine dorsal root ganglia satellite glial cells represent an exceptional cell population with astrocytic and oligodendrocytic properties. Sci. Rep..

[33] Negishi T., Ishii Y., Kawamura S., Kuroda Y., Yoshikawa Y. (2002). Cryopreservation of brain tissue for primary culture. Exp. Anim..

[34] Pischedda F., Montani C., Obergasteiger J., Frapporti G., Corti C., Rosato Siri M., Volta M., Piccoli G. (2018). Cryopreservation of Primary Mouse Neurons: The Benefit of Neurostore Cryoprotective Medium. Front. Cell Neurosci..

[35] Borlongan C.V., Tajima Y., Trojanowski J.Q., Lee V.M., Sanberg P.R. (1998). Transplantation of cryopreserved human embryonal carcinoma-derived neurons (NT2N cells) promotes functional recovery in ischemic rats. Exp. Neurol..

[36] Uemura M., Ishiguro H. (2015). Freezing behavior of adherent neuron-like cells and morphological change and viability of post-thaw cells. Cryobiology.

[37] Edamura K., Nakano R., Fujimoto K., Teshima K., Asano K., Tanaka S. (2014). Effects of cryopreservation on the cell viability, proliferative capacity and neuronal differentiation potential of canine bone marrow stromal cells. J. Vet. Med. Sci..

[38] Hancock C.R., Wetherington J.P., Lambert N.A., Condie B.G. (2000). Neuronal differentiation of cryopreserved neural progenitor cells derived from mouse embryonic stem cells. Biochem. Biophys. Res. Commun..

[39] Ladewig J., Koch P., Endl E., Meiners B., Opitz T., Couillard-Despres S., Aigner L., Brustle O. (2008). Lineage selection of functional and cryopreservable human embryonic stem cell-derived neurons. Stem. Cells.

[40] Taupin P. (2009). Cryopreservation of early postmitotic neuronal cells in culture. Expert Opin. Ther. Pat..

[41] Ichikawa J., Yamada R.X., Muramatsu R., Ikegaya Y., Matsuki N., Koyama R. (2007). Cryopreservation of granule cells from the postnatal rat hippocampus. J. Pharmacol. Sci..

[42] Mattson M.P., Kater S.B. (1989). Development and selective neurodegeneration in cell cultures from different hippocampal regions. Brain Res..

[43] Robert M.C., Juan de Paz L., Graf D.A., Gazzin S., Tiribelli C., Bottai H., Rodriguez J.V. (2016). Cryopreservation by slow cooling of rat neuronal cells. Cryobiology.

[44] Seggio A.M., Ellison K.S., Hynd M.R., Shain W., Thompson D.M. (2008). Cryopreservation of transfected primary dorsal root ganglia neurons. J. Neurosci. Methods.

[45] Negishi T., Ishii Y., Kawamura S., Kuroda Y., Yoshikawa Y. (2002). Cryopreservation and primary culture of cerebral neurons from cynomolgus monkeys (*Macaca fascicularis*). Neurosci. Lett..

[46] Kay G.W., Oswald M.J., Palmer D.N. (2006). The development and characterisation of complex ovine neuron cultures from fresh and frozen foetal neurons. J. Neurosci. Methods.

[47] Hashimoto A., Onodera T., Ikeda H., Kitani H. (2000). Isolation and characterisation of fetal bovine brain cells in primary culture. Res. Vet. Sci..

[48] Cai R.S., Xue D.L., Jiang X.H. (1993). Cryopreservation and culture of the human fetal brain tissues. J. Tongji Med. Univ..

[49] Mattson M.P., Rychlik B. (1990). Cell culture of cryopreserved human fetal cerebral cortical and hippocampal neurons: Neuronal development and responses to trophic factors. Brain Res..

[50] Silani V., Pizzuti A., Strada O., Falini A., Buscaglia M., Scarlato G. (1988). Human neuronal cell viability demonstrated in culture after cryopreservation. Brain Res..

[51] Paynter S.J. (2008). Principles and practical issues for cryopreservation of nerve cells. Brain Res. Bull.

[52] Swett J.W., Paramore C.G., Turner D.A. (1994). Quantitative estimation of cryopreservation viability in rat fetal hippocampal cells. Exp. Neurol..

[53] Andersen N.D., Srinivas S., Pinero G., Monje P.V. (2016). A rapid and versatile method for the isolation, purification and cryogenic storage of Schwann cells from adult rodent nerves. Sci. Rep..

[54] Andres K.H. (1961). Untersuchungen über den Feinbau von Spinalganglien. Zeitschrift für Zellforschung und Mikroskopische Anatomie.

[55] Roloff F., Ziege S., Baumgärtner W., Wewetzer K., Bicker G. (2013). Schwann cell-free adult canine olfactory ensheathing cell preparations from olfactory bulb and mucosa display differential migratory and neurite growth-promoting properties in vitro. BMC Neurosci..

[56] Usoskin D., Furlan A., Islam S., Abdo H., Lonnerberg P., Lou D., Hjerling-Leffler J., Haeggstrom J., Kharchenko O., Kharchenko P.V. (2015). Unbiased classification of sensory neuron types by large-scale single-cell RNA sequencing. Nat. Neurosci..

[57] Bonfanti L., Bellardi S., Ghidella S., Gobetto A., Polak J.M., Merighi A. (1991). Distribution of five peptides, three general neuroendocrine markers, and two synaptic-vesicle-associated proteins in the spinal cord and dorsal root ganglia of the adult and newborn dog: An immunocytochemical study. Am. J. Anat..

[58] Molliver D.C., Wright D.E., Leitner M.L., Parsadanian A.S., Doster K., Wen D., Yan Q., Snider W.D. (1997). IB4-binding DRG neurons switch from NGF to GDNF dependence in early postnatal life. Neuron.

[59] Heng B.C., Ye C.P., Liu H., Toh W.S., Rufaihah A.J., Yang Z., Bay B.H., Ge Z., Ouyang H.W., Lee E.H. (2006). Loss of viability during freeze-thaw of intact and adherent human embryonic stem cells with conventional slow-cooling protocols is predominantly due to apoptosis rather than cellular necrosis. J. Biomed. Sci..

[60] Mattson M.P., Dou P., Kater S.B. (1988). Outgrowth-regulating actions of glutamate in isolated hippocampal pyramidal neurons. J. Neurosci..

[61] Mattson M.P., Kater S.B. (1988). Isolated hippocampal neurons in cryopreserved long-term cultures: Development of neuroarchitecture and sensitivity to NMDA. Int. J. Dev. Neurosci..

[62] Techangamsuwan S.A., Enzo D., Cundi O., Imbschweiler I., Gerhauser I., Wewetzer K., Baumgärtner W. (2011). Cell-cell Interaction Appears to Represent a Pivotal Factor for Susceptibility of Adult Canine Schwann Cell-like Brain Glia to Canine Distemper Virus Infection in vitro. Thai J. Vet. Med..

[63] Bottenstein J.E., Sato G.H. (1979). Growth of a rat neuroblastoma cell line in serum-free supplemented medium. Proc. Natl. Acad. Sci. USA.

[64] Ziege S., Baumgärtner W., Wewetzer K. (2013). Toward defining the regenerative potential of olfactory mucosa: Establishment of Schwann cell-free adult canine olfactory ensheathing cell preparations suitable for transplantation. Cell Transplant..

